# Sensory Quality Evaluation of Superheated Steam-Treated Chicken Leg and Breast Meats with a Combination of Marination and Hot Smoking

**DOI:** 10.3390/foods10081924

**Published:** 2021-08-19

**Authors:** Woo-Hee Cho, Jae-Suk Choi

**Affiliations:** 1 Seafood Research Center, Industry-Academic Cooperation Foundation, Silla University, Busan 46958, Korea; ftrnd3@silla.ac.kr; 2 Department of Food Biotechnology, College of Medical and Life Sciences, Silla University, Busan 46958, Korea

**Keywords:** chicken meat, sensory evaluation, superheated steam, marination, hot smoking, storage effect

## Abstract

As the sensory qualities of meat processed using methods such as superheated steam, marination, and hot smoking have not been examined, this study analyzed the sensory quality of chicken meats (leg, breast) and its chemical correlation by determining optimal processing conditions (superheated steam treatment, marination, and hot smoking). Chicken meats were defrosted using room temperature, running tap water, or high-frequency defroster. Marinated meats with herbal extract solution were treated with superheated steam and then hot smoked with wood sawdust; sensory evaluations were performed at each processing step. The products were analyzed for fatty acids and nutrients, along with storage tests under different conditions. High-frequency defrosting showed the lowest drip loss and thawing time compared to other methods. Bay leaves and oak wood were selected as the best sub-materials for higher sensory scores. Optimal superheated steam conditions showed higher overall acceptance (8.86, 8.71) and were set as follows; leg meat (225 °C; 12 min 20 s), breast meat (223 °C; 8 min 40 s). The final meat products possessed good nutritional composition and no severe sensory spoilages were detected during storage despite microbial and chemical degradations. Thus, regular sensory evaluations at each processing step and storage condition were effective for developing superior chicken meat products.

## 1. Introduction

Chicken meat is a representative major food ingredient that provides good sensory quality as well as nutritional composition and is used almost globally except in certain religions such as Buddhism. Specifically, chicken meat is high in protein and low in fat; in particular, it does not contain trans-fats [1]. Recently, several chicken meats have been processed to prepare completely cooked products to be served as home meal replacements so that consumers can simply consume them regardless of place and time. For example, smoked chicken meat, chicken sausage, and fried chicken are the commonly marketed chicken meat products. For developing high-quality food products, it is very important to establish the optimal processing conditions for maximizing the initial qualities of products, considering the different times required for delivering to the actual consumer. Processes involving high-frequency thawing are reported to minimize the disadvantages of un-optimized thawing methods that typically result in larger ice crystals [2]. Superheated steam treatment results in good sensory improvement like moist texture and decreases both the processing time and microbial activity [3]. Further, marination improves the meat attributes by masking odor [4], and hot smoking provides desirable sensory properties such as flavor and aroma to foods [5] as well as inhibits microorganisms [6].

Several quality analysis methods including microbial, physicochemical, and sensory analyses have been used to evaluate food quality. Among these properties, sensory properties have been recently measured using instruments such as color meter, electronic nose, sodium meter, and texture analyzer. Nevertheless, intuitive sensory evaluation by human senses is remarkably effective, as all sensory parameters should be evaluated comprehensively rather than individually. In reality, numerous studies regarding sensory characteristics have used universal properties such as overall acceptance, which consider all indexes (e.g., appearance, odor, taste, and texture) simultaneously [7].

So far, few studies have examined the sensory qualities of processed meat simultaneously treated with methods such as superheated steam, marination, and hot smoking. Therefore, in this study different processing methods were applied to develop high-quality chicken meat (leg and breast) products; methods including high-frequency thawing, superheated steam treatment, marination, and hot smoking were combined based on previous results. Further, the present study focused on the effect of storage on the qualities of the processed chicken leg and breast, and investigated chemical mechanism related to its sensory results, along with the nutritional composition analysis including fatty acids and basic nutrients.

## 2. Materials and Methods

### 2.1. Sample Preparation

**Figure 1:**
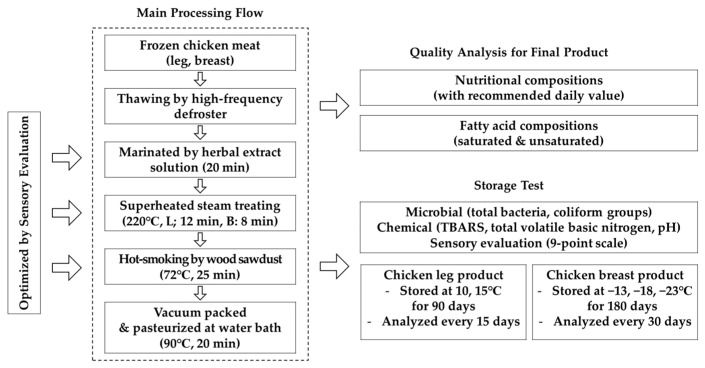
Schematic representation of the development of chicken leg and breast products: L, leg and B, breast.

Chicken leg and breast (Harim Corp., Iksan, Korea) meats were obtained raw from Busan Poultry Cooperative (Busan, Korea) and processed based on the development scheme outlined in Figure 1. Each sample was grouped by similar size [leg; 115–135 (126.4 ± 9.8) mm (W), 54–58 (55.9 ± 1.8, 53.5 ± 2.1) mm (H, T), and breast; 130–140 (136.3 ± 4.2) mm (W), 50–60 (55.7 ± 4.6) mm (H), 34–38 (36.1 ± 1.8) mm (T)] and weight [leg; 90–100 (97.9 ± 5.3) g, and breast; 135–155 (144.2 ± 8.2) g], respectively. All chicken meats were stored at freezing temperature (−18 ± 3 °C) and defrosted using the following thawing methods: room temperature (RT; 15 ± 1 °C in an incubator as per HACCP standards; JSMI-04C, JC Research, Gongju-si, Korea), under running tap water (RW; 23 ± 1 °C), and with high-frequency defrosting (HFD; 27 MHz and 11 kW input power; TEMPERTRON FRT-10, Yamamoto Vinita Co. Ltd., Osaka, Japan).

All herbs (bay leaves, coriander powder, fennel whole, thyme whole, cumin seeds, basil whole, basil powder, and star anise) used for the hot water extract as marination solution were purchased from Solpyo Foods (Gyeonggido, Namyangjusi, Korea) except for sea buckthorn fruit powder, which was obtained from Hub-in-Korea (Gyeonggido, Gimposi, Korea). All marination solutions were prepared by boiling in hot water (100 °C) for 20 min with packaging in a non-woven fabric filter bag at 3% (*w*/*v*). The hot water extract solutions were used after being cooled to RT. Oak, apple, chestnut, walnut, and cherry wood sawdust sticks for hot smoking treatment were purchased from Shinsei (Shinsei Sangyo Co., Tokyo, Japan).

### 2.2. Drip Loss Measurement

Drip loss was measured by comparing the weights of the frozen and defrosted unit samples with each thawing method. All frozen samples and plastic bags (17.7 cm × 18.8 cm; Ziploc for frozen, SC Johnson Korea Ltd., Seoul, Korea) were individually weighed using an electronic scale (MW-2N, CAS Ltd., Yangju-si, Korea), packed at as low-in-air conditions as possible, and defrosted at the same indoor temperature (20 ± 1 °C). After being fully defrosted, the weight of each plastic bag was measured without the defrosted chicken part, which was held as a drip. The drip loss was expressed as the percentage of the initial weight.

Drip loss (%) = [(plastic bag weight after defrosting (g) − original plastic bag weight (g))/frozensample weight (g)] × 100.

### 2.3. Preparation for Treatment

For marination, the defrosted chicken leg and breast meat were immersed in herbal extract solutions containing additional 5% (*w*/*v*) saline for 20 min and were dried on a perforated stainless steel rack at RT for 10 min. Marinated chicken samples were heated using a superheated steam roaster (DFC-560A-2R/L, Naomoto Corporation, Osaka, Japan) under the meat-specific heating condition and treated with wood stick smoke using a hot smoker (Braii Smoker, Bradley, Canada) at 72 °C (Combustion temperature; 250–350 °C) for 25 min considering the conditions of Tirtawijaya et al. [8] and Jeffe et al. [9]. The processed samples were then cooled at RT until the core temperature decreased to below 50 °C and then packaged in vacuum plastic bags. The final products were pasteurized for 20 min in a water bath at 90 °C.

### 2.4. Experiment for Optimization of Superheated Steam Treatment

Superheated steam treatment conditions for chicken leg and breast were optimized using response surface methodology (RSM). Table 1. shows each heating condition for the central composite design (CCD) including independent variables and coded ranges (−1.414, −1, 0, +1, +1.414). The treating temperature (X_1_) and time (X_2_) for both chicken parts were set as respective independent variables. The response surface models of each treatment were derived with the response results, which were obtained with different code combinations, using MINITAB 18 (Minitab Inc., State College, PA, USA). As the response result, the dependent variable (overall acceptance) was measured by a 9-point scale evaluation for sensory analysis. The model was represented as a function of the independent variables using the following quadratic Formula (1):

(1) $$Y=\beta_{0}+\overset{k}{\underset{i=1}{\sum }}\beta_{i}X_{i}+\overset{k}{\underset{i=1}{\sum }}\beta_{\mathrm{ii}}X_{i}^{2}+\overset{k-1}{\underset{i=1}{\sum }}\overset{k}{\underset{j=2}{\sum }}\beta_{\mathrm{ij}}X_{i}X_{j}$$

where β_0_, β_i_, β_ii_, and β_ij_ are the regression coefficients for intercept, linear, quadratic, and interaction terms of the model, respectively. The optimal conditions for superheated steam treatment to chicken leg and breast were obtained through statistical evaluation of the model by analysis of variance (ANOVA) and their actual validation.

### 2.5. Sensory Evaluation

Sensory evaluation (appearance, odor, taste, texture, and overall acceptance) of all samples was done by 21 trained panelists belonging to the Industry-Academic Foundation at Silla University (Busan, Korea) based on a 9-point scale. The result was rated with the following score standards: 9 (best quality), 5 (acceptable limit), and 1 (worst quality).

### 2.6. Microbial Analysis

The microbial quality of the processed product was analyzed by testing the total bacteria count (TBC) and total coliform group (TCG) parameters, respectively. The respective tests were measured in triplicate in accordance with the Association of Official Analytical Chemists (AOAC) 990.12 [10] and 991.14 [11] using rehydratable dry-film media (Aerobic Count Plates and E. coli/Coliform Count plates; 3M, Saint Paul, MN, USA).

### 2.7. Thiobarbituric Acid Reactive Substances

Thiobarbituric Acid Reactive Substances (TBARS) testing was conducted according to the methods described by Yildiz [12] and Mohibbullah et al. [13]. Each sample homogenized with 20% trichloroacetic acid (TCA) in 2M phosphoric acid solution was filtered and incubated with 0.005M thiobarbituric acid for 30 min in a water bath at 95 °C. The sample was cooled to RT and absorbance was measured at 530 nm using a SPECTROstar Nano microplate reader (BMG Labtech, Ortenberg, Germany).

### 2.8. Total Volatile Basic Nitrogen

Total volatile basic nitrogen (TVBN) was measured using the Conway microdiffusion method based on the procedure described by the Ministry of Food and Drug Safety (MFDS), Korea [14]. The sample solution dispensed in a Conway chamber was incubated at 37 °C for 90 min. To quantify TVBN, 0.01 N NaOH was titrated with reactive substances (0.01 N H_2_SO_4_) in the chamber, mixed with Brunswick reagent beforehand.

### 2.9. Hydrogen Ion Concentration (pH)

The pH values of processed chicken leg and breast were measured using an OHAUS Starter 3100 pH meter coupled with a glass electrode (Ohaus, Seoul, Korea) complying with the method described by the MFDS [15].

### 2.10. Analysis of Nutritional Quality

Nutritional compositions (moisture, ash, salinity, calories, sodium, carbohydrates, sugars, dietary fiber, crude fat, trans fat, saturated fat, cholesterol, crude protein, calcium, iron, potassium, and vitamin D) of the processed chicken leg and breast were quantitatively analyzed in line with AOAC 925.09, 923.03, 979.09, 962.09, and 923.05 [16].

### 2.11. Fatty Acid Analysis

For analyzing the fatty acid compositions of the processed chicken leg and breast, a gas chromatograph (GC) (GC-2010 Plus, Shimadzu Corp., Kyoto, Japan) equipped with a flame ionization detector (FID) was used as described in the AOAC 963.22 [17]. After lipid extraction with ether treatment and methylation, fatty acids were separated using the GC-MS column (100 m × 0.25 mm × 0.25 μm; Supelco Inc., Bellefonte, PA, USA) at an oven temperature of 240 °C. The fatty acid values were computed by comparing retention times with standard components.

### 2.12. Storage Quality Analysis

To evaluate the shift in the storage quality of processed chicken leg and breast, the microbial (TBC and TCG), chemical (TBARS, TVBN, and pH), and sensory (appearance, odor, taste, texture, and overall acceptance) indexes were analyzed at different storage conditions (leg; at 10 °C and 15 °C for 90 days, breast; at −13 °C, −18 °C, and −23 °C for 180 days) based on the commonly marketed temperature and shelf-life periods for the specific meat groups [18].

### 2.13. Statistical Analysis

Except for the optimization of superheated steam treatment, all experimental values were measured in triplicate and were analyzed by one-way ANOVA and *t*-tests using SPSS version 23.0 (IBM Corp., Armonk, NY, USA). Statistical significance was set at a *p*-value < 0.05.

## 3. Results and Discussion

### 3.1. Effect of High-Frequency Defrosting on Variation in Drip Loss

**Figure 2:**
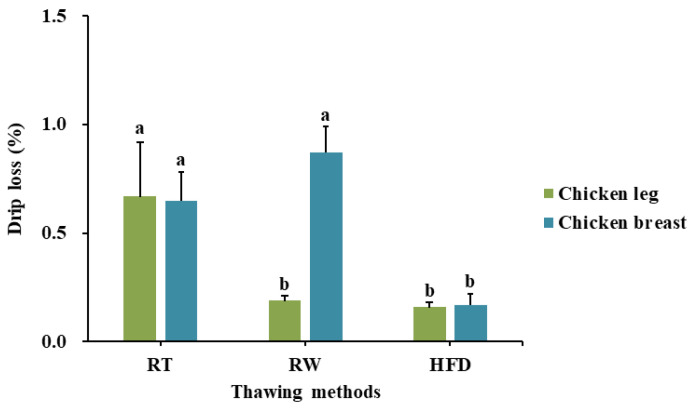
Comparison of drip loss percentage in defrosted samples using different thawing methods (RT, room temperature; RW, running tap water; HFD, high-frequency defroster). Values are mean ± SE. Different letters (a,b) in each column indicate significant differences among the means by Tukey’s test (*p* < 0.10).

The drip loss results of defrosted chicken leg and breast using different thawing methods are shown in Figure 2. The drip loss of defrosted chicken leg indicated that samples thawed with RW and HFD prepared samples showed significantly lower drip loss compared to those thawed at RT, but showed no significant difference between each other. A significantly lower drip loss was observed in the only chicken breast sample that was defrosted using HFD, followed by RT and RW. Overall, the HFD method had a significantly higher effect on decreasing drip loss variation in each chicken part compared to the RT treatment. Furthermore, the chicken breast also showed a significantly effective decrease in drip loss when defrosted by HFD compared to the other methods studied (*p* < 0.10).

In terms of thawing time, each method required different lengths of time for defrosting the samples completely, although they showed similar results by parts: RT (10 h), RW (120 min), and HFD (20 min). Generally, thawing under running tap water was faster than under still air such as at room temperature due to a higher coefficient of heat transfer [19]. In HFD, defrosting by a high-frequency electrode is accelerated because of molecular friction in the muscle cells by a high frequency electrode, which differs from the traditional defrosting methods using thermal conduction. Specifically, the RW and HFD methods exhibited a similar drip loss level in the chicken leg part; however, it was more effective to use HFD considering the thawing time. In contrast, HFD-treated chicken breast samples showed better improvement in terms of both thawing time and drip loss level compared to those treated with RT and RW.

HFD appeared to decrease the drip loss level of both meats more than the other method. In other words, HFD enabled the majority of water inside the foods to be absorbed into the tissue and shortened the time for maximum ice crystal formation using internal heating and balanced melting [20].

The short thawing time and low drip loss level exhibited by HFD can improve quality parameters such as color or hardness as reported in a previous study [21] that examined the same thawing conditions (RT, RW, HFD). Thus, the HFD method was proved to be an optimal thawing method for the preparation of each raw material (chicken leg and breast) considering both the thawing time and drip loss. It can also be effective for maintaining good product quality and minimizing the variables that negatively affect the subsequent sensory evaluation steps.

### 3.2. Optimization of Superheated Steam Treatment of Chicken Leg and Breast

Table 2 shows the response results (Y_L_ and Y_B_; Overall acceptance) from different samples treated with the already defined superheated steam treatment conditions (X_1_; Temperature, X_2_; Time) for chicken leg and breast. Based on these results, the regression equations for the response model were respectively computed as follows:

Y_L_ = 8.573 + 0.2084 X_1_ − 0.1196 X_2_ − 0.549 X_1_X_1_ − 0.501 X_2_X_2_ − 0.380 X_1_X_2_ (2)

Y_B_ = 8.540 + 0.351 X_1_ + 0.230 X_2_ − 1.083 X_1_X_1_ − 0.570 X_2_X_2_ − 0.093 X_1_X_2_ (3)

The analyzed coefficients with a significant effect on each response and their correlations are presented in Table 3. Both the *R*^2^ and *p*-values of the respective models (Y_L_ and Y_B_) satisfied the common recommendation (>0.8) in the previous studies [22] and the statistical standards (<0.05), respectively. Lack of fits revealed inappropriate correlations or the inclusion of considerable factors such as interaction and quadratics, which were also fulfilled the standard (>0.05) in this study. This also indicates that the respective models were suitable for deriving the optimal conditions of superheated steam treatment for chicken leg and breast products.

**Figure 3:**
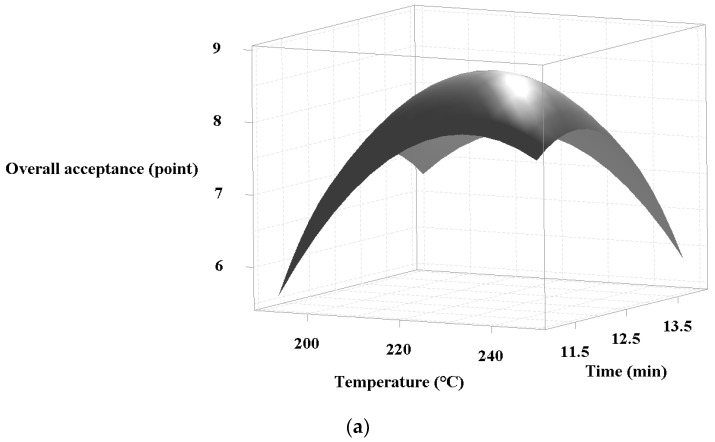

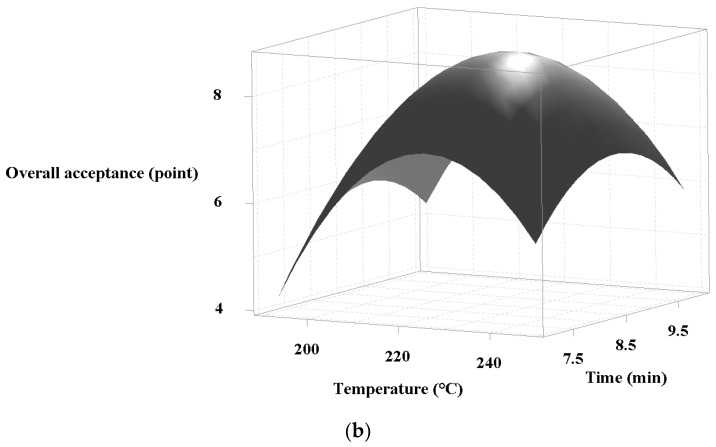
Three-dimensional response surface plots of superheated steam treated chicken products for overall acceptance with respect to temperature and time of roasting: (**a**) chicken leg, (**b**) chicken breast.

Considering the coefficient affecting the responses by the models, both quadratics along with the interaction of Y_L_ showed a significant influence on the results (*p* < 0.05), whereas the linear effects were not valid (*p* > 0.05). Figure 3a,b show the visualized response model for superheated steam treatment of chicken leg and breast, respectively, in a three-dimensional form. The graphs formed a convex curve as they were set to closely central conditions on account of the quadratic impact.

Subsequently, the models showed the maximum response values (overall acceptance) of 8.61 and 8.59 individually. In relation to the optimal range of factors (temperature and time), each model appeared to show almost the same temperature (about 220 °C) whereas the time differed (about 12.5 and 8.5 min for leg and breast, respectively). In general, bone-in meat requires a longer heating time to cook compared to de-boned parts as the bone contributes to slowing heat transfer. Similarly, the cooking time using oven was about two times higher in bone-in pork than in bone-less one in the study of Zilmmermann [23]. In the present study, the chicken leg and breast treated at temperatures lower than 220 °C for less than 12.5 and 8.5 min were not fully cooked and had a few pink spots. However, higher temperatures and longer treatment times resulted in overcooked products, with burned surfaces.

As described in Table 4, the optimal superheated steam treatment conditions for chicken leg and breast were completely determined by the method of maximizing the model responses to each coded variable and validating them with actual experimental results. The models exported 8.61 and 8.59 of the predicted responses (P) when the factors were set to the optimum conditions, respectively; therefore, the experimental samples of chicken leg and breast were respectively prepared at 225 °C for 12 min 20 s and at 223 °C for 8 min 40 s to practically set the equipment. The obtained values (E) were 8.86 and 8.71, and E/P values indicating the error level of prediction were calculated as 1.03 and 1.01 for chicken leg and breast with a high desirability, respectively (>0.9). Further, these completed conditions were continually employed in the next processing steps in this study.

These results were found to be different from those of a previous study [3] in which the sensory evaluation results show that, the best superheated steam-treating conditions of chicken breast fillet are high steam temperature (350 °C) and short heating time (6 min). However, in the present study, the conditions were optimized with lower temperatures and longer times than those in the previous study, and resulted from different sample sizes. In addition, sliced samples were used in the previous study. Therefore, it indicates that the different optimal superheated steam-treating conditions for processing chicken breast also could be confirmed depending on such differences. On the other hand, no studies on superheated steam-treated chicken leg have been described, thus highlighting the importance of the present work.

### 3.3. Effect of Marination with Herbal Extract Solutions on Sensory Evaluation

**Figure 4:**
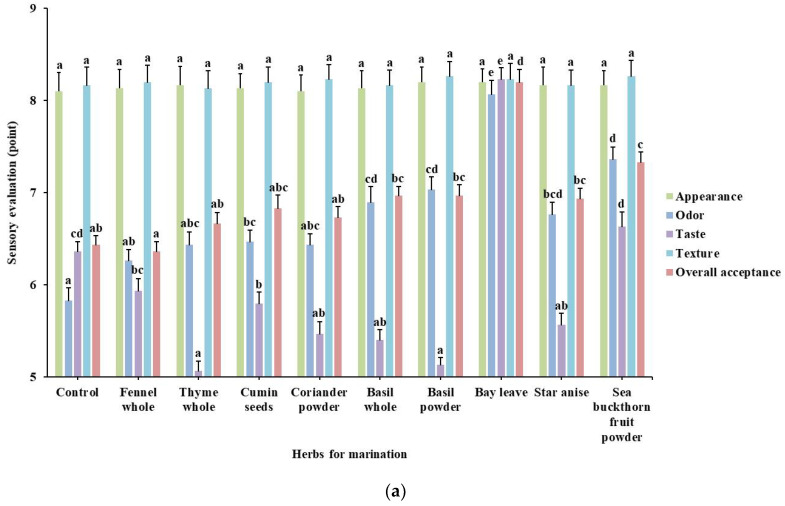

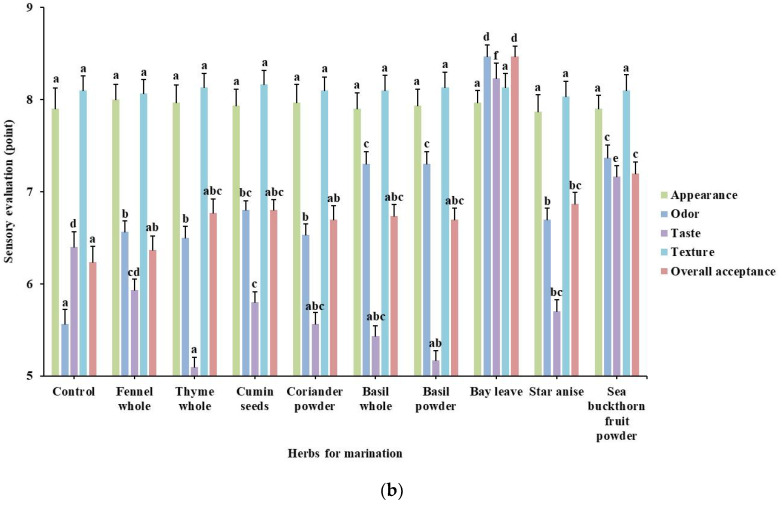
Sensory evaluation of marinated chicken leg and breast with different herbal extract solutions: (**a**), chicken leg; (**b**), chicken breast. Values are mean ± SE. Different letters (a–f) in the respective colored column indicate significant differences among the means by Tukey’s test (*p* < 0.05).

The results of sensory evaluation for determining the optimal herbal extract solution are represented in Figure 4. Herein, bay leaves were established as the optimal herb for marinating both chicken meats and similar score patterns were obtained for both. Specifically, the groups with significantly improved overall acceptance score compared to the control among chicken leg meats were those with bay leaves- and sea buckthorn fruit powder-treatment. In addition, the scores of chicken breast groups were significantly increased in star anise-treated groups, even above those of the other two groups (*p* < 0.05). Interestingly, for chicken breast in particular, the odor score in every marinated group was significantly higher compared to the control one as opposed to the leg. However, no group showed significant variations in appearance and texture indexes.

These results are consistent with those of previous studies about the relationship between herbs and sensory changes. Kurup et al. [24] showed that among the selected herbs and spices, bay leaves, thyme, and coriander have the properties of deodorizing/masking. The study also reported that a harsh and bitter taste from spices was considered to have resulted from the presence of alkaloids, glycosides, and organic and inorganic salts. Lee and An [25] reported that a traditional beef dish with basil added showed decreased taste scores in sensory tests compared to the control group.

### 3.4. Hot Smoking Treatment

**Figure 5:**
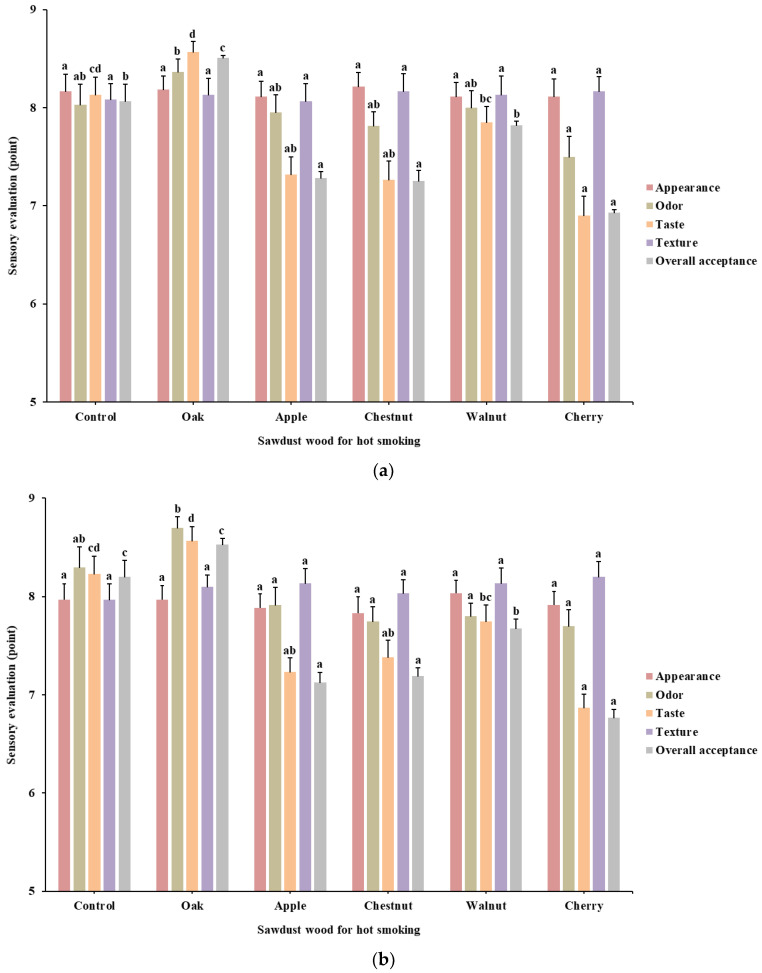
Sensory evaluation of hot-smoked chicken leg and breast with different sawdust: (**a**), chicken leg; (**b**), chicken breast. Values are mean ± SE. Different letters (a–d) in the respective colored column indicate significant differences among the means by Tukey’s test (*p* < 0.05).

The superheated steam-treated and marinated chicken meats were then hot smoked using different wood sawdust as the final processing step, and the results are shown in Figure 5. The results indicated that oak wood had the highest overall score among the sawdust sources used for both meats, and its values were significantly higher than those of other groups including the control group (*p* < 0.05). Further, all the groups did not show a significant difference in the appearance and texture, similar to the marination results. Interestingly, except for the oakwood-treated group, the overall acceptance, odor and taste scores of other groups (apple, chestnut, walnut, and cherry) were lower compared to the control group. In particular, apple, walnut, and cherry wood smoke-treated groups were rated to have significantly reduced points among the sources used (*p* < 0.05).

These different score changes between smoke wood types might be attributed to the different amounts of flavoring contents. Migdał et al. [26] reported that the fruit wood, especially that of apple trees, is rich in hemicellulose, and Ratnani and Widiyanto [27] reported that a fruity and sweet flavor substances such as furfural and furans are produced from the decomposition of hemicellulose. However, in the present study, the chicken meat products were not harmonized with hot-smoking using fruit wood in the described conditions, because it was assumed that the high fruit aroma and taste would cover the essential flavors of chicken meats.

### 3.5. Fatty Acid Analysis

The fatty acid composition profiles of processed chicken leg and breast are shown in Table 5. The results indicated that the processed chicken leg possessed outstandingly higher total fatty acid amount (12.37 g/100 g) than the chicken breast (3.57 g/100 g). The different total fatty acid amounts were derived from the nutritional characteristics of each muscle. Among saturated fatty acids (SFA), palmitic acid in both the leg and breast meat accounted for over two thirds of the fatty acid groups at 3.04 g (/100 g) and 0.88 g (/100 g), respectively. Further, oleic acid was the most abundant fatty acid (5.33 g/100 g and 1.44 g/100 g) among monounsaturated fatty acids (MUFA), as well as total fatty acids, in both the leg and breast part. Among polyunsaturated fatty acids (PUFA), linoleic acid (1.87 g/100 g and 0.52 g/100 g) was the most abundant in both meats. Kralik et al. [28] reported that the dominant SFAs in chicken fat were palmitic and stearic acids whereas unsaturated fatty acids mainly include oleic, linoleic, and arachidonic acids.

Wood et al. [29] mentioned that meat firmness is affected by the melting point of fatty acids in foods, indicating that melting point is decreased with increasing 18C fatty acids such as oleic acid. Similarly, oleic acid increases the perception of juiciness and meat-like flavor [30]. In this study, the processed chicken meats were shown to have good texture and flavor through the sensory evaluation of both leg and breast meat, receiving more softening evaluations in the leg compared to the breast. Another previous study reported that the chicken meat having different fatty acid compositions showed different sensory evaluation results in both the leg and breast [31].

### 3.6. Nutritional Value

The nutritional compositions of processed chicken leg and breast are presented in Table 6. Comparing both products with each other, the chicken leg product showed higher amounts of calories, fats, and cholesterol compared to the chicken breast. Chicken thighs including leg are known to be fattier than the breast and are also moister in comparison [32]. Koh et al. [33] studied the nutrition of chicken depending on the meats and showed that the highest fat amount was included in the wing part, followed by the thigh and breast. Additionally, unlike livestock meat such as beef and lamb, there is no trans-fat in chicken meat [1].

Table 6 shows the respective daily value percentage of each processed product based on the recommended nutrition facts by the FDA. Herein, chicken breast provides a higher amount of protein amount than chicken leg considering unit calories. In case of sodium levels, both meats were measured to keep about 10% DV; however, they revealed different salty tastes compared to each other in actual evaluation, as the saltiness of chicken leg tended to be more concentrated on the surface rather than the inside and the panelists reported feeling a higher salty taste with their first bite. Richter [35] reported that the skin disrupts salt penetration into the meat. According to Tompkin [36], dark meat contains more iron than white meat. In reality, among the nutrition lists, iron content was higher in the processed chicken leg part than in the breast part.

### 3.7. Effect of Storage Conditions on Processed Chicken Meats

Different chicken meat products treated with optimized superheated steam conditions and the best combination of herbal marination and hot-smoking were packaged and tested for each marketed storage condition to investigate the effect of storage on sensory qualities along with microbial and physicochemical parameters.

#### 3.7.1. Sensory Qualities

Table 7 and Table 8 show the sensory results for processed chicken legs and breasts, respectively. At different storage temperatures on chicken leg croups, the parameters that changed the fastest in chicken leg groups were odor at 10 °C and taste at 15 °C, respectively. Both of these were the most significantly decreased during the storage period (*p* < 0.05). The sensory panelists reported that the chicken leg showed slightly unusual and stale smell as storage time increased, and these were more serious in samples stored at 15 °Cthan in those stored at 10 °C. Li et al. [37] reported that the production of off-flavor and odor in meat products is attributed to volatile compounds owing to the metabolites produced by spoilage microorganisms during storage. These could be strongly related to changed TBC and TVBN levels as shown in subsequent results.

Chicken legs stored at both 10 °C and 15 °C showed a significant score decrease in appearance comparing the initial and final day groups (*p* < 0.05). This change was mainly investigated as the result of a color change to a bluish hue according to panelist comments. Katiyo et al. [38] reported that the formation of deoxymyoglobin could affect the change in meat to blue color. In this study, the vacuum packaging formed an oxygen-free condition, which was considered to cause such a change.

When it comes to the significant changes in the texture of processed chicken products during storage periods, fat content plays an important role in determining the texture and tenderness for meat. Kilcast and Lewis [39] said that the crystals surrounded by liquid are formed inside lipid cells at chilling temperatures. In a study by Pande and Akoh [40], larger crystals and softer texture quality or mouthfeel was reported in the final product. Likewise, the chill-storage chicken legs in this study appeared to show a similar tendency with such studies, indicating that the fattier meat of chicken leg became over-soft and inelastic as time passed during the periodical sensory evaluation.

In contrast to chicken leg products, in Table 8. there were few significant sensory degradations in the processed chicken breast during the storage periods. In particular, the samples stored at −23 °C were rated to have almost no significant score changes in all sensory parameters during storage periods, whereas a slight exterior degradation was measured in samples stored at −13 °C, which would be confirmed as a result of the color change to a bluish hue. The samples stored at −13 °C were also found to show significantly decreased taste after the storage period compared to other groups. Different frozen storage conditions could play a crucial role in the taste of foods when defrosted because of dripping loss. Specifically, slow freezing makes the water crystals inside food larger as they slowly pass through a maximum ice crystal formation zone. When they are defrosted for serving and re-heated, they produce a drip that includes important nutrition and tasty compounds. According to Olsson et al. [41], drips decrease the quality of the flesh because of the loss of such desirable components.

#### 3.7.2. Microbial Qualities

The present study analyzed the values of TBC and TCG for processed chicken legs and breasts during each storage condition as shown in Table 9. TBC values were significantly increased for chicken leg stored at 15 °C than for chicken leg stored at 10 °C; similarly, processed chicken breast exhibited higher TBC at a higher storage temperature than at other lower storage temperatures. However, in the whole period, TBC contents were gradually increased in the leg meat products, whereas those of breast meat fluctuated but were maintained between 1.0–1.5 Log CFU/g for 180 days. The latter case could be explained by the inhibition of microorganisms resulting from the oxygen-free condition of vacuum packaging and frozen storage conditions [42]. Overall, no TCG was detected in both meat products for all storage periods and the TBC values did not exceed the acceptable limit (<5 Log CFU/g). This result might be strongly affected by superheated steam treatment at high temperatures, which is consistent with a similar study [43] in which superheated steam-treated chicken skin showed a greater decrease in the number of *Listeria innocua* (CLIP 20595) compared to the sample without superheated steam treatment. Moreover, hot-smoking treatment seems to cause chemically induced inhibition by phenolic compounds as well as thermal inhibition using smoke. Heiszler et al. [44] reported that the increasing surface temperature caused by smoking treatment led to phenol and formaldehyde deposition, which then improved bacteriostatic and bactericidal effects.

#### 3.7.3. Physicochemical Qualities

**Figure 6:**
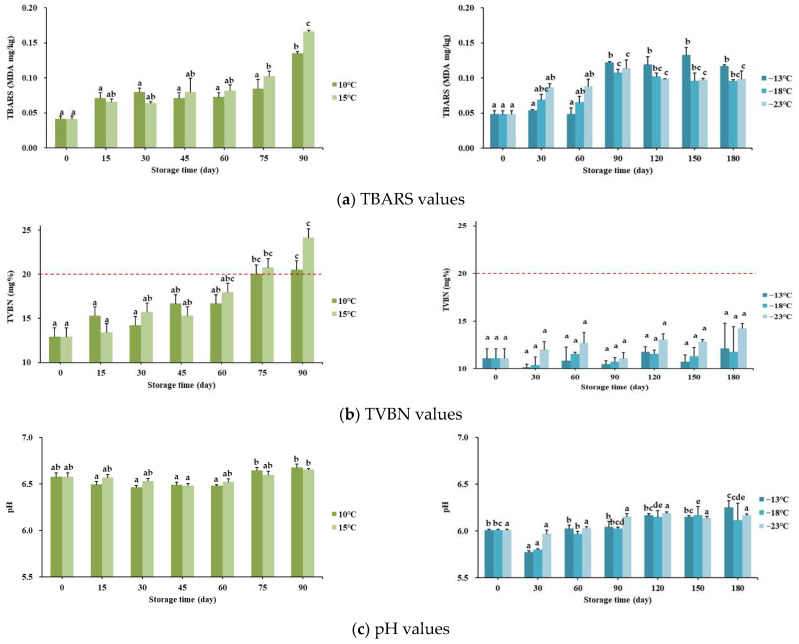
Periodical values in the chemical properties (TBARS, TVBN, pH) of processed chicken products stored at different temperatures for storage days (90 and 180). Values are mean ± SE. Different letters (a–e) indicate significant differences among means by Tukey’s test (*p* < 0.05); ■■ values for chicken leg, ■■■ values for chicken breast.

Figure 6 shows the periodical values in the chemical responses in the processed chicken leg and breast at each storage condition. The values of TBARS for each product are included in Figure 6a. Both processed meats showed change trends similar to those of microbial testing, demonstrating that the TBARS values in chicken leg tended to be steadily increased whereas those in chicken breast were irregularly increased. These values were significantly increased in the final storage period compared to the initial periods (*p* < 0.05).

Domínguez et al. [45] reported that the acceptable limit of TBARS value is 2–2.5 MDA/kg, and suggested that meat and meat products do not become rancid within this standard limit. Likewise, our sensory panelists actually perceived neither severe bitterness nor rancidity, which was the corresponding result, considering that the experimental values remained low.

Figure 6b shows the TVBN levels of processed chicken meats during the storage periods. Comparing both chicken meats at each final storage period, the leg product showed 20.53 ± 0.12 and 24.15 ± 0.70 mg% at 10 °C and 15 °C, whereas the breast product showed 12.13 ± 2.64, 11.78 ± 2.63, and 14.23 ± 0.51 mg% at −13 °C, −18 °C, and −23 °C, respectively. Several previous studies and government food institutes have established the permissible limit of TVBN for fresh meat as follows: 15 mg% [46,47], 20 mg% [48]. Thus, the present study confirmed that chicken legs stored for less than 75 days did not exceed the recommended limit whereas all chicken breast samples satisfied the standard limits.

Related to muscle protein decomposition, higher TVBN values could have an important sensory meaning in the following changes. First, the higher TVBN contributes to the off-flavor of meat owing to the rise in byproducts such as acid compounds and mineral nitrogen [49]. Second, higher TVBN values provokes a rotten and ammonium odor from foods [50]. These sensory responses were similarly observed in the actual sensory test.

As the final indicator in this experiment, pH values cross-reflect chemical reaction by other spoilage factors and are shown in Figure 6c. In this study, we observed that processed chicken leg meat had higher pH values than chicken breast meat. In detail, the former showed pH 6.4–6.7 whereas the latter showed pH 5.7–6.3. From the previous studies on cooked chicken meats, the following results have been assembled. First, chicken thigh has higher pH levels by about 0.5 compared to breast meat [51]. Second, superheated steam-treated chicken breast showed about pH 5.8 in the studies by Choi et al. [52] and Chun et al. [53]. Our results were mostly consistent with these studies.

## 4. Conclusions

With the purpose of developing preferable chicken products, the present study examined the effects of high frequency thawing, determined the optimal superheated steam treatment conditions and sub-materials (marination herb and smoke wood) and compared the storage effect of the final products on the subsequent sensory evaluation by well-trained panelists. Superheated steam treatments for leg and breast meat were optimized at 225 °C for 12 min 20 s and at 223 °C for 8 min 40 s, respectively, showing the best sensory qualities. For better flavor and taste, bay leaf extract was employed for marination of each meat and oak wood was selected as the best hot-smoking sawdust. The final products possessed excellent nutritional composition with balanced fatty acids between PUFA and SFA. Furthermore, both processed chicken leg and breast showed well-maintained sensory qualities scoring over 7 points at each storage condition; however, microbial and chemical degradation of the leg meat product was observed when stored under chilling conditions. Thus, the regular and diversified sensory evaluations related to the chemical correlation at each processing step and storage condition were effective for develop superior chicken leg and breast products.

## Figures and Tables

**Table 1:** Independent variables, codes, and actual levels for optimizing the superheated treatment of chicken products.

Independent Variables	Symbol	Unit	Meats	Range Level				
				−1.414	−1	0	+1	+1.414
Temperature	X_1_	°C	Leg	192	200	220	240	248
			Breast	192	200	220	240	248
Time	X_2_	min	Leg	11.1	11.5	12.5	13.5	13.9
			Breast	7.1	7.5	8.5	9.5	9.9

**Table 2:** Central composite design of independent variables and response of dependent variables during optimization of superheated treatment of chicken products: X_1_, temperature; X_2_, time; Y_L_, overall acceptance of chicken leg; Y_B_, overall acceptance of chicken breast.

Run No.	Coded Values		Chicken Leg			Chicken Breast		
			Actual Values		Responses	Actual Values		Responses
	X_1_	X_2_	X_1_	X_2_	Y_L_	X_1_	X_2_	Y_B_
1	−1	−1	200	11.5	6.95	200	7.5	6.10
2	1	−1	240	11.5	7.90	240	7.5	6.57
3	−1	1	200	13.5	7.67	200	9.5	7.14
4	1	1	240	13.5	7.10	240	9.5	7.24
5	−1.41	0	192	12.5	7.14	192	8.5	5.71
6	+1.41	0	248	12.5	8.05	248	8.5	7.29
7	0	−1.41	220	11.1	8.00	220	7.1	7.48
8	0	+1.41	220	13.9	7.38	220	9.9	7.57
9	0	0	220	12.5	8.38	220	8.5	8.81
10	0	0	220	12.5	8.67	220	8.5	8.33
11	0	0	220	12.5	8.67	220	8.5	8.48

**Table 3:** Analysis of variance for the response of dependent variables during optimization of superheated treatment of chicken products: X_1_, temperature; X_2_, time; Y_L_, overall acceptance of chicken leg; Y_B_, overall acceptance of chicken breast.

Responses	*p*-Value				
	Model	Linear (X_1_, X_2_)	Quadratic (X_1_X_1_, X_2_X_2_)	Interaction (X_1_X_2_)	Lack of Fit
Y_L_ (*R*^2^ = 0.908)	>0.013	0.123	>0.006	>0.035	0.23
Y_B_ (*R*^2^ = 0.903)	>0.014	0.096	>0.004	0.682	0.193

**Table 4:** Optimum conditions for superheated steam-treated chicken products using response surface methodology.

Responses	Optimum Conditions		Predicted Value (*p*)	Experimental Value (E)	E/P	Desirability
	X_1_	X_2_				
Y_L_	+0.27 (225.43 °C)	−0.21 (12.29 min)	8.61	8.86 ± 0.14	1.03	0.97
Y_B_	+0.16 (223.14 °C)	+0.19 (8.69 min)	8.59	8.71 ± 0.18	1.01	0.93

Values are mean ± SE.

**Table 5:** Fatty acid composition of processed chicken leg and breast.

Fatty Acids	Shorthand	Chicken Leg (100 g)	Chicken Breast (100 g)
Caprylic acid	C8:0	0.00	-
Capric acid	C10:0	0.00	0.00
Lauric acid	C12:0	0.00	0.00
Myristic acid	C14:0	0.07	0.03
Pentadecanoic acid	C15:0	0.02	0.03
Palmitic acid	C16:0	3.04	0.88
Magaric acid	C17:0	0.01	0.01
Stearic acid	C18:0	0.76	0.23
Arachidic acid	C20:0	0.01	0.00
Heneicosylic acid	C21:0	0.01	0.00
Behenic acid	C22:0	0.01	0.00
∑ SFA ^1^		3.94	1.18
Myristoleic acid	C14:1	0.02	0.01
Palmitoleic acid	C16:1	0.93	0.24
Magaoleic acid	C17:1	0.01	0.01
Oleic acid	C18:1	5.30	1.44
Eicosenoic acid	C20:1	0.01	0.00
Eicosadienoic acid	C20:2	0.02	0.01
Erucic acid	C22:1	0.02	0.01
∑ MUFA ^2^		6.33	1.73
Linoleic acid	C18:2 *n*-6	1.87	0.52
γ-Linolenic acid	C18:3 *n*-6	0.02	0.01
Dihomo γ-Linolenic acid	C20:3 *n*-6	0.00	0.00
Arachidonic acid	C20:4 *n*-6	0.07	0.04
∑ *n*-6		1.96	0.57
Linolenic acid	C18:3 *n*-3	0.13	0.04
Eicosatrienoic acid	C20:3 *n*-3	0.00	0.00
Eicosapentaenoic acid (EPA)	C20:5 *n*-3	0.00	0.01
Docosapentaenoic acid (DPA)	C22:5 *n*-3	0.01	0.01
Docosahexaenoic acid (DHA)	C22:6 *n*-3	0.00	0.03
∑ *n*-3		0.15	0.09
∑ PUFA ^3^		2.10	0.66
Total fatty acid (g)		12.37	3.57
PUFA/SFA		0.53	0.56
*n*-6/*n*-3		13.07	6.33

^1^ SFA: saturated fatty acid. ^2^ MUFA: monounsaturated fatty acid. ^3^ PUFA: polyunsaturated fatty acid.

**Table 6:** Nutritional values of processed chicken leg and breast.

Parameters		Chicken Leg (100 g)		Chicken Breast (100 g)		Daily Values ^1^
		Amount	% DV ^2^	Amount	% DV ^2^	
Ash	g	1.31	-	1.50	-	-
Calories	cal	241.46	-	149.26	-	-
Sodium	g	0.22	9.6	0.24	10.4	2.3
Carbohydrate	g	0.65	0.2	1.18	0.4	275
Sugar	g	0.62	1.2	1.00	2.0	50
Dietary fiber	g	1.46	5.2	2.16	7.7	28
Crude fat	g	12.46	16.0	3.58	4.6	78
Trans fat	g	-	-	-	-	2
Saturated fat	g	3.94	19.7	1.18	5.9	20
Cholesterol	mg	138.63	46.2	75.53	25.2	300
Crude protein	g	25.83	51.7	28.08	56.2	50
Vitamin D	μg	-	-	-	-	20
Potassium	g	0.28	6.0	0.32	6.8	4.7
Iron	mg	5.27	29.3	3.18	17.7	18
Calcium	g	0.15	11.5	0.08	6.2	1.3

^1^ According to Nutrition Facts Labeling Requirements, US Food and Drug Administration. ^2^ The % daily value (DV) indicates how much a nutrient in a serving of food contributes to the daily diet. Normally, 2000 calories a day is used in general nutrition advice. Reprinted from US Food and Drug Administration (2020) [34].

**Table 7:** Periodical values of sensory evaluation of processed chicken leg stored at different temperatures (10 °C, and 15 °C) for 90 days.

Temp	Day	Appearance	Odor	Taste	Texture	Overall Acceptance
10 °C	0	8.48 ^a^ ± 0.15	8.29 ^a^ ± 0.16	8.52 ^a^ ± 0.13	8.24 ^a^ ± 0.15	8.52 ^a^ ± 0.11
	15	8.43 ^a^ ± 0.13	8.19 ^a,b^ ± 0.15	8.48 ^a^ ± 0.13	8.24 ^a^ ± 0.14	8.62 ^a^ ± 0.13
	30	8.33 ^a^ ± 0.16	8.05 ^a,b^ ± 0.20	7.95 ^a,b^ ± 0.15	8.43 ^a^ ± 0.16	8.38 ^a^ ± 0.15
	45	8.24 ^a^ ± 0.14	7.86 ^a,b,c^ ± 0.16	7.90 ^a,b^ ± 0.14	8.05 ^a,b^ ± 0.18	8.14 ^a^ ± 0.14
	60	8.10 ^a^ ± 0.12	7.48 ^b,c^ ± 0.18	7.86 ^a,b^ ± 0.26	8.00 ^a,b^ ± 0.20	8.10 ^a^ ± 0.19
	75	7.19 ^b^ ± 0.13	6.71 ^d^ ± 0.20	7.33 ^b^ ± 0.21	7.29 ^c^ ± 0.16	7.33 ^b^ ± 0.14
	90	7.19 ^b^ ± 0.16	7.24 ^c,d^ ± 0.21	7.48 ^b^ ± 0.18	7.48 ^b,c^ ± 0.19	7.48 ^b^ ± 0.13
15 °C	0	8.48 ^a^ ± 0.15	8.29 ^a^ ± 0.16	8.52 ^a^ ± 0.13	8.24 ^a^ ± 0.15	8.52 ^a^ ± 0.11
	15	8.29 ^a^ ± 0.12	8.14 ^a^ ± 0.13	8.10 ^a,b^ ± 0.10	8.29 ^a^ ± 0.14	8.29 ^a^ ± 0.12
	30	8.19 ^a^ ± 0.11	8.05 ^a^ ± 0.19	7.90 ^a,b^ ± 0.15	7.81 ^ab^ ± 0.16	8.19 ^a^ ± 0.13
	45	8.10 ^a^ ± 0.12	8.05 ^a^ ± 0.15	7.71 ^b,c^ ± 0.14	8.00 ^a^ ± 0.15	8.05 ^a^ ± 0.13
	60	7.33 ^b^ ± 0.17	6.86 ^b^ ± 0.16	7.14 ^c^ ± 0.19	7.24 ^b^ ± 0.14	7.29 ^b^ ± 0.14
	75	7.14 ^b^ ± 0.13	6.90 ^b^ ± 0.17	7.10 ^c^ ± 0.17	7.24 ^b^ ± 0.15	7.05 ^b^ ± 0.13
	90	7.05 ^b^ ± 0.15	6.90 ^b^ ± 0.18	7.19 ^c^ ± 0.19	7.24 ^b^ ± 0.12	7.19 ^b^ ± 0.15

Values are mean ± SE. Different letters (a–d) in each column indicate significant differences among means by Tukey’s test (*p* < 0.05).

**Table 8:** Periodical values of sensory evaluation in processed chicken breast stored at different temperatures (−13 °C, −18 °C, and −23 °C) for 180 days.

Temp	Day	Appearance	Odor	Taste	Texture	Overall Acceptance
−13 °C	0	8.48 ^a^ ± 0.13	8.14 ^a^ ± 0.13	8.33 ^a^ ± 0.14	8.29 ^a^ ± 0.16	8.48 ^a^ ± 0.13
	30	8.43 ^a,b^ ± 0.11	8.10 ^a^ ± 0.12	8.19 ^a,b^ ± 0.13	8.05 ^a^ ± 0.15	8.10 ^a,b^ ± 0.15
	60	8.10 ^a,b,c^ ± 0.18	7.71 ^a,b^ ± 0.17	7.95 ^a,b^ ± 0.16	8.14 ^a^ ± 0.17	8.24 ^a,b^ ± 0.15
	90	8.10 ^a,b,c^ ± 0.18	7.90 ^a,b^ ± 0.14	8.05 ^a,b^ ± 0.19	7.86 ^a^ ± 0.17	8.19 ^a,b^ ± 0.15
	120	7.76 ^a,b,c^ ± 0.19	7.43 ^b^ ± 0.11	7.76 ^a,b^ ± 0.15	7.76 ^a^ ± 0.15	8.05 ^a,b^ ± 0.13
	150	7.67 ^b,c^ ± 0.21	7.67 ^a,b^ ± 0.19	7.90 ^a,b^ ± 0.17	7.57 ^a^ ± 0.20	7.81 ^b^ ± 0.16
	180	7.62 ^c^ ± 0.22	7.57 ^a,b^ ± 0.16	7.57 ^b^ ± 0.13	7.57 ^a^ ± 0.20	7.76 ^b^ ± 0.15
−18 °C	0	8.48 ^a^ ± 0.13	8.14 ^a^ ± 0.13	8.33 ^a^ ± 0.14	8.29 ^a^ ± 0.16	8.48 ^a^ ± 0.13
	30	8.24 ^a,b^ ± 0.14	8.10 ^a^ ± 0.12	8.14 ^a^ ± 0.16	8.24 ^a^ ± 0.14	8.43 ^a^ ± 0.15
	60	8.48 ^a^ ± 0.11	8.00 ^a^ ± 0.14	8.10 ^a^ ± 0.18	8.05 ^a^ ± 0.16	8.33 ^a^ ± 0.13
	90	8.29 ^a,b^ ± 0.17	7.81 ^a,b^ ± 0.15	8.10 ^a^ ± 0.18	8.19 ^a^ ± 0.18	8.38 ^a^ ± 0.15
	120	7.62 ^b^ ± 0.19	7.29 ^b^ ± 0.17	7.62 ^a^ ± 0.16	7.62 ^a^ ± 0.18	7.90 ^a^ ± 0.15
	150	8.05 ^a,b^ ± 0.20	8.00 ^a^ ± 0.18	7.62 ^a^ ± 0.18	7.81 ^a^ ± 0.21	8.14 ^a^ ± 0.19
	180	8.10 ^a,b^ ± 0.17	7.90 ^a,b^ ± 0.15	7.95 ^a^ ± 0.21	7.90 ^a^ ± 0.18	8.05 ^a^ ± 0.16
−23 °C	0	8.48 ^a^ ± 0.13	8.14 ^a^ ± 0.13	8.33 ^a^ ± 0.14	8.29 ^a^ ± 0.16	8.48 ^a^ ± 0.13
	30	8.24 ^a^ ± 0.15	8.10 ^a^ ± 0.17	8.10 ^a,b^ ± 0.14	8.19 ^a^ ± 0.18	8.38 ^a^ ± 0.15
	60	8.19 ^a^ ± 0.18	8.00 ^a^ ± 0.17	8.00 ^a,b^ ± 0.18	7.86 ^a^ ± 0.16	7.95 ^a^ ± 0.15
	90	7.76 ^a^ ± 0.19	7.71 ^a^ ± 0.20	8.00 ^a,b^ ± 0.15	7.81 ^a^ ± 0.20	8.14 ^a^ ± 0.19
	120	7.90 ^a^ ± 0.17	7.76 ^a^ ± 0.19	7.52 ^b^ ± 0.19	7.57 ^a^ ± 0.20	7.86 ^a^ ± 0.16
	150	7.76 ^a^ ± 0.17	7.67 ^a^ ± 0.16	7.76 ^a,b^ ± 0.19	7.71 ^a^ ± 0.14	7.90 ^a^ ± 0.15
	180	7.86 ^a^ ± 0.19	7.71 ^a^ ± 0.20	8.00 ^a,b^ ± 0.18	7.57 ^a^ ± 0.16	7.95 ^a^ ± 0.18

Values are mean ± SE. Different letters (a–c) in each column indicate significant differences among means by Tukey’s test (*p* < 0.05).

**Table 9:** Periodical values in the total bacterial count (TBC; Log CFU/g) of processed chicken products stored at different temperatures for different storage days (90 and 180).

Day	Chicken Leg		Day	Chicken Breast		
	10 °C	15 °C		−13 °C	−18 °C	−23 °C
0	2.53 ^a^ ± 0.04	2.53 ^a,b^ ± 0.04	0	ND	ND	ND
15	1.78 ^a^ ± 0.36	2.29 ^a^ ± 0.02	30	1.48 ^a^ ± 0.44	1.00 ± 0.00	ND
30	2.02 ^a^ ± 0.30	2.43 ^a^ ± 0.08	60	1.30 ± 0.00	1.00 ± 0.00	1.40 ± 0.00
45	1.84 ^a^ ± 0.45	2.73 ^b,c^ ± 0.04	90	1.09 ^a^ ± 0.12	1.15 ^a^ ± 0.21	1.20 ^a^ ± 0.28
60	2.11 ^a^ ± 0.25	2.77 ^b,c^ ± 0.10	120	1.39 ^a^ ± 0.30	1.35 ^a^ ± 0.07	1.24 ^a^ ± 0.34
75	2.14 ^a^ ± 0.47	2.88 ^c^ ± 0.12	150	1.36 ^a^ ± 0.22	1.15 ^a^ ± 0.21	1.16 ^a^ ± 0.28
90	2.84 ^a^ ± 0.17	3.43 ^d^ ± 0.04	180	1.62 ^a^ ± 0.28	1.30 ^a^ ± 0.30	1.24 ^a^ ± 0.34

Values are mean ± SE. Different letters (a–d) in each column indicate significant differences among the means by Tukey’s test (*p* < 0.05).

## Data Availability

Data supporting reported results are available upon request.
